# The golden era of biomedical informatics has begun

**DOI:** 10.1186/s13040-016-0092-6

**Published:** 2016-04-11

**Authors:** Jason H. Moore, John H. Holmes

**Affiliations:** Department of Biostatistics and Epidemiology, Institute for Biomedical Informatics, Perelman School of Medicine, University of Pennsylvania, Philadelphia, PA 19104-6116 USA

## Abstract

Biomedical informatics has become a central focus for many academic medical centers and universities as biomedical research because increasingly reliant on the processing, analysis, and interpretation of large volumes of data, information, and knowledge. We posit here that this is the beginning of the golden era of biomedical informatics with opportunity for this maturing discipline to have a substantial impact on the biggest questions and challenges facing efforts to improve human health and the healthcare system.

The American Medical Informatics Association (AMIA) defines biomedical informatics as an “interdisciplinary field that studies and pursues the effective uses of biomedical data, information, and knowledge for scientific inquiry, problem solving, and decision making, motivated by efforts to improve human health” [1]. The discipline is inclusive of areas such as bioinformatics and computational biology, clinical informatics, clinical research informatics, consumer health informatics, and public health informatics. The history of the discipline as it relates to healthcare in the United States has been described in great detail in the recent book edited by Collen and Ball [2].

Biomedical informatics has become a central focus for many academic medical centers and universities as biomedical research because increasingly reliant on the processing, analysis, and interpretation of large volumes of data, information, and knowledge. We posit here that this is the beginning of the golden era of biomedical informatics with opportunity for this maturing discipline to have a substantial impact on the biggest questions and challenges facing efforts to improve human health and the healthcare system. We briefly review below several reasons for this. Some of these will be expanded upon in future editorials.

First, we have entered the era of big data with substantial volumes of measurements (variables and/or observations) from new technology such as high-throughput DNA sequencing and wearable devices that capture real-time physiological signals. Everyone from biochemists to emergency room healthcare providers are awash in data that can be paralyzing in both size and complexity. Biomedical informatics is uniquely positioned to provide the skillset and the technology for processing, managing, retrieving, analyzing, and interpreting big data from both the basic and clinical sciences. Informatics methods such as natural language processing (NLP) and technology such as graph databases have the potential to unlock large sets of unstructured data that often dwarf sources of data that have a predefined structure that are easier to work with. Further, the open-data movement is making many different sources of big data accessible to the masses. This will greatly improve the impact of data that has been sometimes expensive, time-consuming, and even impossible to collect. Access to big data comes with important governance challenges that are just now being experienced by those working in biomedical informatics.

Second, high-performance computing (HPC) technology is now available and inexpensive, thus enabling sophisticated computational analyses of big data. Local resources such as parallel computing clusters are relatively inexpensive to set up and consume a much smaller footprint in data centers as CPUs are loaded with more and more cores. Computing using graphic processing units (GPUs) is also becoming mainstream as the video game industry has motivated faster and faster graphics capability. Each GPU now has thousands of cores that are enabled by better and better libraries for adapting code to harness these miniature HPC resources that can often fit under a desk. Global HPC has never been better with large companies such as Amazon, Google, and Microsoft now competing to offer researchers access to their cloud-based HPC resources. Third-party companies are now springing up to offer data security layers for cloud computing that makes it possible to use these resources for data with personal health information (PHI). Open science grids are also emerging to allow free access to computers on the internet. Such powerful computing has never been cheaper and more accessible.

Third, artificial intelligence (AI) and machine learning have matured and are now in some cases performing at or beyond human capacity with challenges such as playing Jeopardy or Go. The impressive performance by intelligent algorithms is partly due to the more than 50 years of research on fundamental methods such as NLP and neural networks and partly due to the availability of powerful HPC resources. IBM was able to put all of these pieces together for their Watson question-answering product that is now being marketed to academic medical centers for enabling precision medicine, for example. This is a turning point for AI and many see the AI winter that resulted from premature hype to be over. There is no question that AI has an important role to play in biomedical research and healthcare. The challenge for the golden era will be to bring this technology to the masses in such a way that does not over-promise, but makes manifest the tangible benefits of AI in the biomedical domain.

Fourth, visualization and, more specifically, visual analytics are ready for integration across the discipline of biomedical informatics. This is especially true given the volume and complexity of both research and healthcare data. Information visualization is made possible by hardware tools that include inexpensive high-definition televisions and off the shelf software tools and libraries for programming languages, such as D3, a JavaScript package for producing data visualizations in web browsers. Improvements in human-computer interaction are rapidly progressing, with new hardware for touch and gesture computing and innovative software that facilitates both virtual and augmented reality. We have recently commented in an editorial on the role that visualization and novel technology such as 3D printing can play in biomedical research [3]. As with AI, our challenge is to bring this technology to the masses as smartphone manufacturers and game developers have.

Fifth, biomedical informaticians are in the unique position of being able to integrate many different knowledge sources that can be used to inform scientific questions that have the potential to be more impactful than those formulated using knowledge specific to a single discipline. We have referred to this in previous papers as no-boundary-thinking (NBT) in informatics [4, 5]. The NBT approach is focused on letting knowledge drive the question-asking process. This is in contrast to recent trends whereby big data are used to define questions that are inspired only by nuances of the data. For example, genomic medicine can now measure genetic variation across the entirety of the human genome. It is commonplace to test for genetic associations one DNA sequence variant at a time treating each as an independent factor. While this approach embraces the big data, it completely ignores the pathobiology of the disease that is captured in numerous knowledge sources such as PubMed. The NBT approach defines questions from knowledge and then seeks to identify the appropriate data, big or small, to answer the question. The challenge for the golden era is the management of this knowledge and the metadata resources, so that the appropriate data can be identified and used effectively.

Sixth, the practice of biomedical informatics as a research discipline has matured such that informaticians are now seen as leaders of collaborative research projects. In the early days of the discipline, biomedical informaticians were seen as consultants or service personnel that were called upon to help with a data management or data analysis need at one point in the scientific method. This quickly changed as it became apparent that informaticians were needed throughout the research endeavor as true collaborators. The golden era will see informaticians leading research projects by asking scientific questions and establishing teams of experts to answer those questions. The central role of informaticians as the drivers of scientific projects is partly due to the increased focus on interdisciplinary training and the widespread availability of data, information, and knowledge that can be harnessed using an NBT approach. At the same time, informaticians are increasingly holding their own as key personnel on research projects, to the extent that they are becoming as essential as statisticians to a successfully funded grant. The tension between the roles of independent researcher and co-investigator illustrates a challenge for the profession that is nothing short of existential.

Seventh, there is increasing recognition among private, federal, and academic institutions of the value of biomedical informatics. For example, the pharmaceutical industry is hiring informaticians as quickly as we can train them. This has made the market more competitive driving up salaries for both staff and faculty. Further, the National Institutes of Health of the United States created in 2014 a new Associate Director for Data Science to help position the NIH to respond to the growing demand for biomedical informatics methods for big data. The role is in addition to the existing National Library of Medicine that represents the biomedical informatics institute at the NIH with both intramural and extramural informatics research programs. Finally, university and academic medical centers are starting new departments, programs, centers, and institutes focused on biomedical informatics at a rapid pace. This expansion of the academic infrastructure for informatics comes at a time when many institutions are struggling in the new economic landscape heavily impacted by a slowing global economy. The investment in biomedical informatics has never been greater. The challenge for the golden era is that informaticians must clearly demonstrate that the discipline’s contributions make this investment worthwhile.

It is difficult to predict the duration of the golden era of biomedical informatics. The demand and the potential impact will remain high for the foreseeable future as research and healthcare become increasingly dependent on data and the synthesis and interpretation of information and knowledge. There is no question that it is a good time to be an informatician.
